# 

**Published:** 1910-05

**Authors:** 


### BOOKS RECEIVED.

Diseases of Infancy and Childhood. By Henry Koplik, M.D., Attending Physician to the Mount Sinai Hospital, New York. Third edition. Octavo, pp. 944. Illustrated. New York and Philadelphia: Lea & Febiger. (Cloth, $5.00.)

A Practical Treatise on Fractures and Dislocations. By Lewis A. Stimson, B.A., M.D., Professor of Surgery in Cornell University Medical College, New York. Sixth edition. Illustrated. Octavo, pp. 876. New York and Philadelphia: Lea & Febiger. (Cloth, $5.00.)

The Optic Nerve and the Accessory Sinuses of the Nose. By Professor A. Onodi, Budapest, Member of the Hungarian Academy of Sciences. Translated by J. Luckoff, M.D., Cape Town. Octavo, pp. 101. With 50 illustrations. New York: William Wood & Co. 1910. (Price, $3.50 net.)

Modern Medicine. Its Theory and Practice. Edited by William Osler, M.D., Regius Professor of Medicine in Oxford University, England. Assisted by Thomas McCrea, M.D., Associate Professor of Medicine and Clinical Therapeutics in Johns Hopkins University. In seven octavo volumes, illustrated. Volume VII. Diseases of the Nervous System. Philadelphia and New York. 1909. Lea & Febiger, Publishers. (Per volume: cloth, $6.00; leather, $7.00; half morocco, $7.50, net prices.)

Prescription Writing and Formulary. By John M. Swan, M.D., Associate Professor of Clinical Medicine, Medico-Chirurgical College of Philadelphia. 32 mo of 185 pages. Philadelphia and London: W. B. Saunders Company, 1910. (Flexible leather, $1.25 net.)

Pocket Therapeutics and Dose-Book. By Morse Stewart, Jr., B.A., M.D. Fourth edition. Small 32 mo. of 263 pages. Philadelphia and London: W. B. Saunders Company, 1910. (Cloth, $1.00 net.)

A Textbook of Pathology. By Joseph McFarland, M.D., Professor of Pathology and Becteriology in the Medico-Chirurgical College of Philadelphia. Second edition. Octavo of 856 pages, with 437 illustrations, some in colors. Philadelphia and London. W. B. Saunders Company, 1910. (Cloth, $5.00; half morocco, $6.50 net prices.)

Duodenal Ulcer. By B. G. A. Moynihan, M.S. (London) F.R.C.S., Senior Assistant Surgeon at Leeds General Infirmary, England. Octavo of 379 pages, illustrated. Philadelphia ad London: W. B. Saunders Company. 1910. (Cloth, $4.00; half morocco, $5.50 net prices.)

